# The Presence of Genotoxic and/or Pro-inflammatory Bacterial Genes in Gut Metagenomic Databases and Their Possible Link With Inflammatory Bowel Diseases

**DOI:** 10.3389/fgene.2018.00116

**Published:** 2018-04-10

**Authors:** Abiel Roche-Lima, Kelvin Carrasquillo-Carrión, Ramón Gómez-Moreno, Juan M. Cruz, Dayanara M. Velázquez-Morales, Igor B. Rogozin, Abel Baerga-Ortiz

**Affiliations:** ^1^Center for Collaborative Research in Health Disparities – RCMI Program, Medical Sciences Campus, University of Puerto Rico, San Juan, Puerto Rico; ^2^Department of Biochemistry, Medical Sciences Campus, University of Puerto Rico, San Juan, Puerto Rico; ^3^Molecular Sciences Research Center, University of Puerto Rico, San Juan, Puerto Rico; ^4^National Center for Biotechnology Information, U.S. National Library of Medicine, Bethesda, MD, United States

**Keywords:** metagenomics, human microbiome, inflammatory bowel diseases, bioinformatics, *usp*, *gelE*, next-generation sequencing data

## Abstract

**Background:** The human gut microbiota is a dynamic community of microorganisms that mediate important biochemical processes. Differences in the gut microbial composition have been associated with inflammatory bowel diseases (IBD) and other intestinal disorders. In this study, we quantified and compared the frequencies of eight genotoxic and/or pro-inflammatory bacterial genes found in metagenomic Whole Genome Sequences (mWGSs) of samples from individuals with IBD vs. a cohort of healthy human subjects.

**Methods:** The eight selected gene sequences were *clbN, clbB, cif, cnf-1, usp, tcpC* from *Escherichia coli, gelE* from *Enterococcus faecalis* and *murB* from *Akkermansia muciniphila*. We also included the sequences for the conserved *murB* genes from *E. coli* and *E. faecalis* as markers for the presence of *Enterobacteriaceae* or *Enterococci* in the samples. The gene sequences were chosen based on their previously reported ability to disrupt normal cellular processes to either promote inflammation or to cause DNA damage in cultured cells or animal models, which could be linked to a role in IBD. The selected sequences were searched in three different mWGS datasets accessed through the Human Microbiome Project (HMP): a healthy cohort (*N* = 251), a Crohn’s disease cohort (*N* = 60) and an ulcerative colitis cohort (*N* = 17).

**Results:** Firstly, the sequences for the *murB* housekeeping genes from *Enterobacteriaceae* and *Enterococci* were more frequently found in the IBD cohorts (32% *E. coli* in IBD *vs.* 12% in healthy; 13% *E. faecalis* in IBD *vs.* 3% in healthy) than in the healthy cohort, confirming earlier reports of a higher presence of both of these taxa in IBD. For some of the sequences in our study, especially *usp* and *gelE*, their frequency was even more sharply increased in the IBD cohorts than in the healthy cohort, suggesting an association with IBD that is not easily explained by the increased presence of *E. coli* or *E. faecalis* in those samples.

**Conclusion:** Our results suggest a significant association between the presence of some of these genotoxic or pro-inflammatory gene sequences and IBDs. In addition, these results illustrate the power and limitations of the HMP database in the detection of possible clinical correlations for individual bacterial genes.

## Introduction

The human-associated microbiota is a diverse and dynamic community of microorganisms that carry out numerous biochemical processes, many of them associated with the maintenance of good health but others associated with diseases such as colorectal cancer and inflammatory diseases (Blaser, 2014; Bonnet et al., 2014; Marchesi et al., 2016). In recent years, there has been an increase in the number of studies aimed at delineating the involvement of the microbiota in the etiology, presentation and frequency of human diseases (Turnbaugh et al., 2006; Karlsson et al., 2013; Blaser, 2014; Sinha et al., 2016). The wide availability of parallel methods for DNA sequencing has made it possible to answer questions on whether the microbial community profile (the composition and distribution of species as measured by 16S rRNA gene) or the gene composition within the microbial community (by shotgun sequencing methods), are associated with disease.

With the goal of elucidating the role of the microbiome in disease, the Human Microbiome Project (HMP) was established in 2008 with the financial backing of the NIH Common Fund (Proctor, 2016). Through this interdisciplinary effort, the microbial communities and metagenomic whole genome sequence (mWGS) datasets from different body sites, were obtained from healthy individuals and from individuals diagnosed with a specific disease, such as Crohn’s disease or ulcerative colitis. The overarching goals of the HMP were to define a core microbiota for healthy individuals and to determine whether variations from this core microbiota could be associated with disease. In order to accomplish this, the HMP carried out the analysis of samples from 242 healthy adults from 15 or 18 body sites up to three times, which generated 5,177 microbial taxonomic profiles from 16S ribosomal RNA genes and over 3.5 terabases of metagenomic sequence (Human Microbiome Project Consortium, 2012a). Additionally, the HMP also sponsored 15 demonstration projects with the goal of testing hypothesized correlations between the microbiome and human disease. These investigator-initiated projects leveraged advances made by the HMP’s large scale sequencing efforts to examine the relationship between changes in the human microbiome and diseases of interest, using 16S rRNA, metagenomic shotgun sequencing or both^1^.

Previous research from our group, rather than focusing on the presence of particular microbial taxa, emphasized on detecting specific gut microbial genes in the human population by PCR analysis of stool samples (Gomez-Moreno et al., 2014). Of particular interest to us were bacterial genes that encode genotoxic or pro-inflammatory factors that have been shown to cause, or correlate with, tissue inflammation either in cell-based assays, animal models or in epidemiological studies. Our hypothesis was that by interrogating a set of bacterial genes, it should be possible to assess the inflammatory status of the GI tract, an indicator of susceptibility to inflammatory bowel diseases or colorectal cancer (Terzic et al., 2010). This set of genes includes the cyclomodulins reported to be in some strains of *E. coli*, such as *cif* (**c**ycle **i**nhibiting **f**actor), cnf-1 (**c**ytotoxic **n**ecrotizing **f**actor) and the *pks* island (which encodes the production of the natural product colibactin) (Nougayrède et al., 2005, 2006). Other genes present in some strains of E. coli, such as *tcpC*, which encodes an inhibitor of toll-like receptors and induces the formation of urinary tract abscesses, and *usp* that encodes the **u**ropathogenic **s**pecific **p**rotein, which is a highly toxic nuclease that causes DNA damage (Yadav et al., 2010; Zaw et al., 2013). The preliminary frequencies for these genotoxic or pro-inflammatory bacterial genes in the general population was determined through a simple PCR analysis of stool samples obtained from a clinical laboratory in Puerto Rico (Gomez-Moreno et al., 2014). Although the sample size in that study was relatively small (*N* = 41), the pathogenic genes were found to be present in frequencies as high as 20%, a figure that is consistent with previous epidemiological analysis carried out on bacterial isolates (Johnson et al., 2008; Arthur et al., 2012).

We now report the search for those genotoxic and/or pro-inflammatory bacterial gene sequences in a select group of HMP mWGS datasets: the HMP healthy cohort and two IBD cohorts from the HMP demonstration projects. We aimed to determine whether any of the genes in our set are found in higher (or lower) numbers in the stool samples from patients with IBDs than in the healthy cohort, as their increased presence would suggest an association with IBD. Our results show that the frequency for many of these genes, notably the *pks island* and *tcpC*, are lower in the HMP database than what is typically reported experimentally by PCR amplification. Also, some of these genes notably *usp* and *gelE*, are found more frequently in IBD cohorts than in the healthy population, indicating a possible role in promoting inflammation in the GI tract. Taken together these results illustrate the power of the HMP databases in the detection of the possible involvement of individual bacterial genes in diseases, while also highlighting the difficulties associated with monitoring gene sequences of low abundance in highly complex samples.

## Materials and Methods

### Gene Selections and Sequence Retrieval

A total of eight genotoxic or pro-inflammatory bacterial genes encoding cyclomodulins, and other gene products were selected for this analysis based on their reported ability to promote inflammation *in vitro* or in animal models. The first set of genes included *clbN* and *clbB* (genes within the *pks island* gene cluster from *Escherichia, Klebsiella, Citrobacter*, and *Enterobacter* species), along with *cif, cnf-1* and *usp*. These genes were selected because their ability to interfere with the cell cycle (Nougayrède et al., 2005). The second set of genes were *tcpC* (from *E. coli*) and *gelE* (from *E. faecalis*), which have been shown to increase inflammation in infected tissues and in animal models (Cirl et al., 2008; Steck et al., 2011; Golinska et al., 2013). Finally, the *murB* gene, a specific marker for the presence of *Akkermansia muciniphila*, was also included in this study because of reports linking this species to colorectal cancer (Weir et al., 2013).

In addition to the genotoxic or pro-inflammatory gene sequences, we also included the sequences for two housekeeping genes: *murB* from *E. coli* and *murB* from *E. faecalis*. These were included in the analysis to account for the relative presence of these species (i.e., *E. coli* and *E. faecalis*) in the samples. We accessed GenBank database (Benson, 2005) from NCBI to acquire the DNA sequences in FASTA format for all the 10 genes (i.e., eight pathogenic pro-inflammatory genes and 2 housekeeping genes). **Table 1** summarizes all the genes in the study.

**Table 1:** Bacterial genes considered in this study.

Gene	Organism	Base pairs	GenBank accession	Clinical correlate
*clbB*	*Escherichia coli strain IHE3034*	9,620	AM229678.1	Part of the pks genomic island – genotoxic
*clbN*	*Escherichia coli strain IHE3034*	4,637	AM229678.1	Part of the pks genomic island – genotoxic
*usp*	*Escherichia coli strain K-12*	1,041	AB027193	Linked with urinary infections
*cnf-1*	*Escherichia coli strain A70.1*	3,044	U42629.1	Linked with uncontrolled cell division
*gelE*	*Enterococcus faecalis*	1,529	D85393.1	Increases intestinal inflammation in mice
*tcpC*	*Escherichia Coli strain S107*	924	GQ902994.1	Formation of kidney abscesses
*cif*	*Escherichia coli strain EF33*	849	AY128544.1	Cell cycle modulation
*AMmurB*	*Akkermansia muciniphila*	678	AM905291.1	Intestinal mucin digestion
**Housekeeping genes**			
*ECmurB*	*Escherichia coli strain K-12*	1,029	NC_000913.3 [Region: 4172057-4173085]	
*EFmurB*	*Enterococcus faecalis strain V583*	903	NC_004668.1 [Region: 2642156-2643058]	

*Columns represent: gene name (grouped as genotoxic or pro-inflammatory and housekeeping genes), Organism (bacteria and strain), Base Pairs (Total number of nucleotides per gene) and GenBank Accession (Identifier of the FASTA gene sequence).*

### Cohort Selections and Gene Hits

Three different mWGS cohorts were selected and accessed through the Human Microbiome Project – Data Analysis and Coordination Center (HMP-DACC) (Human Microbiome Project Consortium, 2012a,b). The first cohort was the HMP healthy cohort, which includes shotgun sequence data from stool samples of 251 healthy individuals directly accessed from HMP-DACC. The second and third cohorts contain sequences that were obtained through the HMP-DACC portal for the Demonstration Project Disease Cohorts (NCBI BioProject Accession PRJNA46305). The Crohn’s Disease cohort contains data extracted from the project “Metagenomic Analysis of the Structure and Function of the Human Gut Microbiota in Crohn’s Disease,” University of Maryland, Baltimore (dbGap Accession phs000257^2^). In this study, mWGS data was obtained from 60 stool samples from patients with Crohn’s Disease. The Ulcerative Colitis cohort included data from the project “The Role of the Gut Microbiota in Ulcerative Colitis” (dbGap Accession phs000262^3^; University of Michigan at Ann Arbor). In this case, mWGS data was obtained from 17 stool samples from Ulcerative Colitis patients. Information on the cohorts is summarized in **Table 2** and all the biosamples used in this study are listed in Supplementary Tables S2–S4.

**Table 2:** Information about the cohorts.

Cohort	Number of samples	Project study	BioProject accession
HMP Healthy	251 (136M + 115F)^a^	HMP-DACC [18,19]	PRJNA43017
Crohn’s Disease	60 (23M + 37F)	Metagenomic Analysis of the Structure and Function of the Human Gut Microbiota in Crohn’s Disease [20]	PRJNA46321
Ulcerative Colitis	17 (16M + 1F)	The Role of the Gut Microbiota in Ulcerative Colitis [21]	PRJNA46881

*This information includes the cohort name, number of samples, name of the project (with reference) and BioProject Accession (project identifier to obtain the shotgun sequence sample data). ^*a*^136 males and 115 females in the healthy cohort.*

In order to obtain the mWGS data of stool samples from the 251 healthy individuals, the following steps were followed:

Step 1: Access the mWGS reads and extract each of their sample accession identifiers (i.e., Sequence Read Archive Sample Accessions – SRSIds);
Step 2: Retrieve the experimental identifiers (i.e., Sequence Read Experiment Accessions – SRXIds), using SRSIds;
Step 3: Use the SRXIds as BLAST parameters to query the corresponding pathogenic or housekeeping gene.

In the case of Crohn’s disease and ulcerative colitis datasets, we had direct access to the SRXIds, available in each project and went directly to Step 3, using BLAST to query the pathogenic and housekeeping genes with SRXIds as BLAST parameters. The BLAST database selected for this analysis was Sequence Read Archive (SRA-BLAST) (Leinonen et al., 2011) and the algorithm was MegaBLAST (Morgulis et al., 2008). The default parameters selected for the MegaBLAST algorithm were: Expected Value = 10, Word size = 28, Reward match = 1, Penalty mismatch = -2, Gap Existence = 0, Gap extension = 2.5, Percent Identity = None, Filtering = Low Complexity and Matrix = Not Applicable for this Algorithm. Gene hits were considered as the BLAST alignment results of a gene in mWGS with a query coverage value greater than 0. The number of Samples with Hits for each gene was the number of samples with at least one hit for a particular gene. The Total Samples with Hits per Cohort was obtained by counting the total number of samples with at least one hit within a cohort. The Frequency for each gene was then computed as the percentage of Samples with Hits divided by the total number of samples in each cohort.

### Statistical Analysis

There are numerous challenges in providing statistical analysis for SRA-BLAST/WGS reads, mainly because of many factors that could confound the final results (for example, differences in size of the datasets, differences in sequencing quality among datasets). We employed a simple framework which tends to produce more reliable results when several variables simultaneously influence a process (Nei and Kumar, 2001; Rogozin and Pavlov, 2003). As a first step, we compared the sizes of metagenomic datasets for healthy and disease cohorts (see Supplementary Table S1) using the two-tail Student *t*-test. Significant differences were found between them: metagenomic datasets for healthy individuals tend to have a substantially larger size (see Supplementary Table S1). Thus, we expected that metagenomic datasets for healthy individuals should be substantially enriched in SRA-BLAST hits. The null hypothesis is that the frequency of samples with SRA-BLAST hits is the same for healthy and affected individuals. In order to test this null hypothesis, we compared raw numbers of samples with SRA-BLAST hits for healthy and affected individuals using the one-tail Fisher exact test. In a different method, a Monte Carlo modification of the Pearson χ^2^ test of spectra homogeneity (Adams and Skopek, 1987) was used to compare raw numbers of samples with SRA-BLAST hits in each gene for healthy and affected individuals. The calculations were done using the COLLAPSE program (Khromov-Borisov et al., 1999).

## Results

### Presence of Genotoxic or Pro-inflammatory Genes in the Healthy Population

The presence of the genotoxic or pro-inflammatory gene sequences in the HMP Healthy cohort is shown in the **Table 3**, Healthy Cohort. The most frequently encountered sequence was *AMmurB* (*murB* from *A. muciniphila*) with 10% frequency, meaning it was present in 10% of the samples from this population. *AMmurB* was closely followed by the *usp* and *tcpC* genes, with 5 and 3% frequencies, respectively. The other bacterial sequences have frequencies of less than 1. In total, genotoxic or pro-inflammatory sequences were found in 16% of individuals in the healthy cohort with 4% harboring more than one of these sequences. We will use this data as a baseline for statistical comparisons with samples from IBD cohorts, i.e., Crohn’s disease and ulcerative colitis cohorts.

**Table 3:** Presence of genotoxic or pro-inflammatory genes of the three cohorts selected for this analysis, i.e., Healthy, Crohn’s disease and Ulcerative colitis.

	Healthy cohort (*N* = 251)		Crohn’s disease cohort (*N* = 60)		Ulcerative colitis cohort (*N* = 17)	
Genes (p-values)^a^	Positives (M/F)	Frequency %	Positives	Frequency %	Positives	Frequency %
*AMmurB* (0.3026)	25 (11M + 14F)^b^	10	4	7	0	0
*usp* (2 × 10^-5^)	13 (8M + 5F)	5	6 (2M + 4F)	10	6 (6M)^d^	35
*tcpC* (0.7031)	7 (4M + 3F)	3	1	2	0	0
*cnf-1* (0.0081)	2 (2F)	0.8	4	7	0	0
*gelE* (2 × 10^-7^)	2 (2M)	0.8	9 (3M + 6F)	15	0	0
*cif* (0.0154)	1 (1F)	0.4	0	0	1	6
*clbB* (0.4978)	1 (1F)	0.4	1	2	0	0
*clbN* (0.0922)	1 (1M)	0.4	2	3	0	0
**Total^b^**	**40*^c^***	**16%**	**19*^c^***	**31%**	**6*^c^***	**29%**

*Columns represent: gene names, number of samples with hits (positives) and the corresponding frequency of samples with gene hits per study cohort. ^*a*^*P*-value for the differences between the number of samples with and without gene hits across the three studied cohorts (Khromov-Borisov et al., 1999). ^*b*^11 males and 14 females positive for the presence of this gene. ^*c*^Total number of individuals positive for at least one gene. Some had more than one gene. ^*d*^The ulcerative colitis cohort consisted of 16 males and one female.*

### Presence of Genotoxic or Pro-inflammatory Genes in Individuals With Crohn’s Disease

The genes *gelE* and *usp* were the most commonly found sequences in the mWGS samples from individuals with Crohn’s disease (15 and 10% frequency, respectively). They are followed by *cnf-1* and *AMmurB* with 7% frequency each. Finally, *clbN, clbB* and *tcpC* genes are less common, with frequencies ranging between 2 and 3%. In this cohort, the *cif* gene was not present. More information about the genes, samples with hits, and frequencies can be seen in **Table 3**.

The total number of individual samples positive for genotoxic or pro-inflammatory gene sequences in the Crohn’s Disease cohort was significantly higher than in the healthy cohort. In total 19 samples were positive for at least one gene in the Crohn’s Disease cohort (31% total frequency) with 8 samples (13%) harboring more than one gene. The analysis reveals a statistically significant difference with *P* = 0.00004 according to the Fisher exact test vs. the healthy cohort (40 samples with hits vs. 211 samples without hits). This result is significant even if we take into account the conservative Bonferroni correction for multiple hits (the threshold *p*-value is 0.025 = 0.05/2). This result suggests that there is a substantial and significant excess of some genotoxic or pro-inflammatory bacterial genes in Crohn’s disease samples, particularly *gelE* and *usp*.

### Presence of Genotoxic or Pro-inflammatory Genes in Individuals With Ulcerative Colitis

The ulcerative colitis cohort also reveals *usp* as the most common pro-inflammatory gene sequence with a frequency of 35%. It is followed by *cif* with 6% frequency. All the other six pathogenic gene sequences were not found in this small cohort of 16 samples.

The total number of individual samples positive for genotoxic or pro-inflammatory gene sequences in the Ulcerative Colitis cohort was higher than in the healthy cohort. In this case, a total of 5 individual samples (out of 17 samples) contained at least one of the gene sequences in our (29% total frequency) versus 16% total frequency in the healthy cohort (**Table 3**). Also, this result is statistically significant with a *P* = 0.016 according to the Fisher exact test and is significant after the Bonferroni adjustment for the number of comparisons (2 in this case). The higher frequency of hits in the ulcerative colitis cohort vs. healthy, mirrors the findings in the Crohn’s disease samples, especially for the *usp*.

### Presence of the Housekeeping Genes in the Three Different Cohorts

We initially intended to use the 16S rRNA gene sequence *of E. coli* as an indicator of the presence of this species in the population. However, very few samples were found to contain this specific sequence. Instead we chose to monitor the presence of sequences for *MurB*, an essential gene for the production of peptidoglycan in both species: *E. coli* and *E. faecalis*. However, *ECMurB* is approximately 80% similar to the *MurB* of other members of the *Enterobacteriaceae* family, such as *Citrobacter, Enterobacter, Klebsiella* etc. Thus, although the detection of this gene may be indicative of the presence of *E. coli*, it may also indicate the presence of related *Enterobacteriaceae*. Similarly, the *EFMurB* was also found to be around 80% similar to the MurB of other related *Enterococcus* species.

The presence of these bacterial housekeeping gene sequences in the mWGS cohorts are shown in **Table 4**. The most common bacterial housekeeping sequence that we found in all cohorts was *ECmurB*, consistent with the generalized prevalence of *Enterobacteriaceae* in the gut microbiota. The gene *murB* from *E. faecalis* is also highly present in the samples. Results show what appears to be an increased presence of *Enterobacteriaceae* and *Enterococci* in the IBD cohorts versus the healthy cohort (**Table 4**).

**Table 4:** Presence of housekeeping genes in the metagenomic sequences for all cohorts.

Cohort/Total of samples	Genes	Samples with hits	Frequency (%)
Healthy/251	*ECmurB*	29	12
	*EFmurB*	8	3
Crohn’s disease/60	*ECmurB*	13	22
	*EFmurB*	12	20
Ulcerative colitis/17	*ECmurB*	7	41
	*EFmurB*	1	6
	*ECmurB*	*P*-value ^a^	0.0011
	*EFmurB*	*P*-value	1.1 × 10^-5^

*Columns represent: cohort name and the total of samples per cohort, gene names, number of samples with gene hits and the corresponding frequency of gene hits. ^*a*^*P*-value for the differences between the number of samples with and without hits for *ECmurB* or *EFmurB* across the three cohorts (Khromov-Borisov et al., 1999).*

This higher frequency of *EFMurB* and *ECMurB* in the IBD samples could reflect the fact that these two bacterial species (*E. faecalis* and *E. coli*) are more abundant in IBD samples than in control and thus, their genes (including the studied pathogenic genes) are also more abundant. In order to test whether the observed association between specific bacterial genes and IBD was only tracking the presence of certain species, we compared the frequency of samples with SRA-BLAST hits for the pathogenic genes and housekeeping genes.

We tested homogeneity (Khromov-Borisov et al., 1999) of tables that contain raw numbers of samples with SRA-BLAST hits in each gene for healthy and affected individuals (Supplementary Table S5). The comparison between *ECmurB* and the genotoxic or pro-inflammatory gene sequences revealed a statistically significant heterogeneity of the table (*P* = 0.0088), a similar result (*P* = 0.0086) was found for the comparison between *EFmurB* and the genotoxic or pro-inflammatory genes (Supplementary Table S6). Analysis of individual genes suggested that certain genes are over-represented in IBD. The *gelE* gene is over-represented in Crohn’s disease Cohort for comparison with *EFmurB* (*P* = 0.00028, the Fisher exact test). The *usp* gene was found with a marginally significantly higher frequency in the Ulcerative Colitis Cohort (*P* = 0.0426, the Fisher exact test) for comparison with *ECmurB*. These results suggest that the presence of genotoxic and/or pro-inflammatory genes in the IBD cohorts is higher than would be expected based on the expansion of their bacterial hosts in these samples.

## Discussion

The advent of parallel sequencing methods and the development of computational tools designed to handle the troves of resulting data, have been instrumental in the growing field of microbiome research. At present, there are many explanations for how the microbiome contributes to human disease and this knowledge has required the implementation of diverse experimental and computational strategies, including systematic searches using the data that has already been generated and deposited in the HMP databases. In this work, we searched for specific bacterial genes in datasets that were generated under the auspices of the HMP. The fact that a common set of protocols was followed for sample collection, preparation and data acquisition, should ensure consistency in the quality and comparability of the data across cohorts. The data analyzed in this work was generated at multiple centers using two different sequencing platforms: the healthy cohort DNA was analyzed on an Illumina GAIIx platform, whereas the DNA from the IBD samples was sequenced on a 454 FLX Titanium platform. There are reported differences between these two platforms in terms of the read length and the total number of reads in a dataset (Luo et al., 2011). However, the HMP protocols were developed to ensure that the data generated using these two platforms are comparable (Human Microbiome Project Consortium, 2012b). Part of the HMP internal validation involved the use of a mock microbial community (HMP Accession No. PRJNA48475) that was analyzed and assembled using both Illumina and 454 sequencing and the ensuing comparisons between the centers and across platforms demonstrated high consistency of target sequencing depth and success rates (Human Microbiome Project Consortium, 2012a). More recently, it has been shown that shotgun metagenomic data obtained from 454 and Illumina platforms result in the identification of 90% of the same genes, suggesting that despite differences in read length and number or reads, the resulting data can be compared (Luo et al., 2011).

In this work, we searched these HMP datasets and demonstration projects for the presence of a subset of bacterial gene sequences that had previously been found to be associated with genotoxicity or with the promotion of inflammation (Gomez-Moreno et al., 2014). We chose bacterial gene sequences purposefully to match the subset of genes for which experimental data was already available (Sifri et al., 2002; Cuevas-Ramos et al., 2010; Nipic et al., 2013; Snyder et al., 2013).

In the healthy cohort, the most abundant sequence was that of *AMmurB* from *Akkermansia muciniphila*, a bacterium previously associated with colorectal cancer (Weir et al., 2013). However, no statistical association with IBD was found for *AMmurB* (*p*-value = 0.3026). For all other genes in this healthy cohort of 251 samples, we found the frequency for all genotoxic or pro-inflammatory bacterial sequences in the databases to be lower than the gene frequency obtained through PCR detection as reported previously (Johnson et al., 2008; Arthur et al., 2012; Gomez-Moreno et al., 2014). For instance, previous studies had shown *pks island* genes to be present in 20% of *E. coli* strains isolated from the mucosa samples of healthy human donors (Arthur et al., 2012). In a separate study, the *pks island* genes were found in 32% of *E. coli* strains isolated from stool samples of hospitalized veterans (Johnson et al., 2008). Also, the same *pks island* genes were present in 20% of stool samples obtained from a local clinical laboratory in Puerto Rico (Gomez-Moreno et al., 2014). Others have documented the prevalence of *pks island* genes in France, Japan, and United Kingdom to be between 15 and 40% (Shimpoh et al., 2017). By contrast, the present study reveals the *pks island* sequences to be present in only one sample out of 251 from healthy individuals in the HMP database (0.4%). The same can be said for the *tcpC* gene, which had been previously found in 17% of stool samples by PCR, yet its sequence was present in just 3% of the samples in the HMP database (Gomez-Moreno et al., 2014). Similarly, *gelE*, a known virulence factor from *E. faecalis*, had been found in higher numbers by PCR analysis of stool samples (7%), than in the HMP database (0.8%). This underrepresentation of bacterial gene sequences of possible clinical relevance in the databases, could be indicative of the challenges associated with the detection of genes that are naturally in low abundance such as virulence factors or enzymes involved in secondary metabolism, both of which have been shown to be underrepresented in this type of dataset (Sansonetti, 2010). By contrast, there is a reported over-representation of high-abundance genes such as those involved in primary metabolism (Sansonetti, 2010). Thus, it is possible that the specific detection and study of low-abundance genes will require the development of new sample preparation or sequencing protocols specific for this purpose.

Despite the very low frequency of detection for some of the genotoxic or pro-inflammatory bacterial gene sequences, there were statistically significant increases in their detection frequency in the IBD cohorts, suggesting a possible link with IBD. The gene *gelE* was found in higher numbers in the Crohn’s disease cohort (15% vs. 0.8% in healthy). Similarly, *usp* was also found more commonly in both Crohn’s and ulcerative colitis cohorts (35% vs. 5% in healthy). The correlation between the presence of *gelE* and IBD has been suggested previously (Steck et al., 2011; Golinska et al., 2013). Studies with mice lacking both copies of the *IL10* gene registered higher levels of intestinal inflammation when infected with strain of *E. faecalis* harboring the *gelE* gene than those infected with a strain lacking *gelE* (Steck et al., 2011). Also, the *gelE* gene was found in higher frequency in isolates of *E. faecalis* obtained from the feces of children with IBD vs. children without IBD (Golinska et al., 2013). It is possible that the presence of gelE protease in the colon could damage the mucosal lining in a way that promotes inflammation by impairing the epithelial barrier, thus exposing cells to the microbial community (Steck et al., 2011). Our current study confirms this association, thus providing some means of validation for the approach of searching for specific bacterial genes in metagenomic databases.

The correlation of *usp* with IBD was more unexpected. The gene *usp* was originally characterized in isolates of uropathogenic *E. coli* (Nakano et al., 2001). Its activity was confirmed to be part of a nuclease containing and H-N-H motif, a structural element important for its function as a cytotoxic bacteriocin (Zaw et al., 2013). However, the role of usp in intestinal strains of *E. coli* remains unknown. In this study, we report a possible correlation between the presence of *usp* in the gut and IBD, possibly through the same cell damage mechanisms that cause it to affect the urinary tract. Further investigations will be required before this correlation can be fully confirmed experimentally in a clinical study, and its mechanism can be delineated.

Our findings of a higher frequency of *E. coli* genes or *E. faecalis* genes in IBD samples vs. healthy controls, could reflect the fact that these two bacterial species are more abundant in IBD samples than in control and thus, their genes are also more abundant. To test this, we assumed that the frequency of the housekeeping genes *ECmurB* and *EFmurB* for both species approximately corresponds to the abundance of *E. coli* and *E. faecalis* bacteria in the different cohorts, although the genes could also be detecting other related species. Clearly there is a higher presence of these species in the samples from IBD cohorts than the healthy cohort, as evidenced by the increased presence of the *murB* housekeeping genes. The association between the presence of *E. coli* and IBD is well established (Martinez-Medina and Garcia-Gil, 2014). Also, *E. faecalis* strains have been shown to modulate inflammation through a MAPK signaling pathway, and this effect is consistent with a possible involvement in IBDs (Wang et al., 2014). However, the results of the statistical analysis indicate that the increase in the presence of genotoxic and/or pro-inflammatory genes in the IBD cohorts (vs. healthy) is significantly higher than the increase in the *E. coli* and *E faecalis murB* genes in the same IBD samples, suggesting that the increased presence of *usp* and *gelE* is not just due to an increased presence of their bacterial hosts.

Overall, we present a strategy for querying the HMP databases in a focused and hypothesis-based manner in search for bacterial genes that may be of clinical interest. Our hypothesis is that preliminary associations with disease may emerge from a search that emphasizes on specific bacterial genes for which the mechanisms of toxicity or tissue damage are known. This search for candidate genes would be greatly facilitated if the vast amounts of data in existence were to be available through a common repository that is both searchable and user-friendly. Efforts such as the United States National Microbiome Initiative constitute a step in the right direction especially if it allows for the establishment of a searchable global microbiome database, a necessary generator of testable hypotheses on the role of the gut microbiota in intestinal diseases (Bouchie, 2016).

## Author Contributions

AR-L designed and led the bioinformatic analysis of the data. AB-O led the team to test the hypothesis and achieve the goals. AR-L and AB-O mostly contributed to the drafting of this manuscript. KC-C, DV-M, RG-M, and JC executed and processed the analysis, from different perspectives. KC-C also participated in the writing of the manuscript. IR performed and wrote the statistical analysis. All the authors reviewed and approved the manuscript.

## Conflict of Interest Statement

The authors declare that the research was conducted in the absence of any commercial or financial relationships that could be construed as a potential conflict of interest. The reviewer CH and handling Editor declared their shared affiliation.

## Footnotes

1 https://www.hmpdacc.org/health/projectdemos.php

2 The data/analyses presented in the current publication are based on the use of study data downloaded from the dbGaP web site, under phs000257.v2.p1 (https://www.ncbi.nlm.nih.gov/projects/gap/cgi-bin/study.cgi?study_id=phs000257.v2.p1).

3 The data/analyses presented in the current publication are based on the use of study data downloaded from the dbGaP web site, under phs000262.v2.p1 (https://www.ncbi.nlm.nih.gov/projects/gap/cgi-bin/study.cgi?study_id=phs000262.v2.p1)
