# Supplementary material for: The Presence of Genotoxic and/or Pro-inflammatory Bacterial Genes in Gut Metagenomic Databases and Their Possible Link With Inflammatory Bowel Diseases
Source: Front Genet. 2018 Apr 10;9:116. doi: 10.3389/fgene.2018.00116 (PMC5902703; doi:10.3389/fgene.2018.00116)
Supplement: Supplementary file 1 [file Data_Sheet_1.docx]

**Supplementary Table S1**.Unpaired t-test of the differences between sizes (Mbases) of metagenomic Whole Genome Sequence for healthy and IBD cohorts.

**Healthy samples vs.Crohn's Disease**

Mean Stand.Dev. Mean Stand.Dev. tdf P

6585 5027 4058 7208 3.2 309 0.0016

------

**Healthy samples vs. Ulcerative Colitis**

Mean Stand.Dev. Mean Stand.Dev. tdf P

6585 5027 2341 1834 3.5 266 0.0006

**Supplementary Table S2**.List of samples in the healthy cohort of the HMP. (Bioproject PRJNA48479 - stool samples)

| **BioSample** | **Experiment** | **Run** | **SRA_Sample** | **sex** | **Mbases** | **Mbytes** |
| --- | --- | --- | --- | --- | --- | --- |
| SAMN00037108 | SRX018273 | SRR038746 | SRS015890 | M | 3929 | 2524 |
| SAMN00040202 | SRX019702 | SRR041654 | SRS018984 | M | 3853 | 2455 |
| SAMN00036649 | SRX019703 | SRR041658 | SRS015431 | M | 3709 | 2388 |
| SAMN00040286 | SRX019704 | SRR041662 | SRS019068 | M | 3820 | 2502 |
| SAMN00035018 | SRX022900 | SRR059338 | SRS013800 | M | 6583 | 3939 |
| SAMN00037803 | SRX022908 | SRR059346 | SRS016585 | F | 7244 | 4506 |
| SAMN00038919 | SRX022914 | SRR059352 | SRS017701 | F | 7051 | 43080 |
| SAMN00039039 | SRX022916 | SRR059354 | SRS017821 | M | 6935 | 41059 |
| SAMN00034433 | SRX022928 | SRR059366 | SRS013215 | M | 6437 | 43443 |
| SAMN00038207 | SRX022934 | SRR059372 | SRS016989 | M | 7436 | 49098 |
| SAMN00038651 | SRX022940 | SRR059378 | SRS017433 | F | 6079 | 39282 |
| SAMN00039569 | SRX022956 | SRR059394 | SRS018351 | M | 6324 | 40387 |
| SAMN00033491 | SRX022974 | SRR059412 | SRS012273 | F | 6593 | 3961 |
| SAMN00034120 | SRX022982 | SRR059420 | SRS012902 | F | 5858 | 39176 |
| SAMN00034694 | SRX023066 | SRR059504 | SRS013476 | M | 6779 | 46459 |
| SAMN00034739 | SRX023201 | SRR059812 | SRS013521 | F | 5150 | 3268 |
| SAMN00038465 | SRX023221 | SRR059828 | SRS017247 | M | 3924 | 2419 |
| SAMN00037971 | SRX023222 | SRR059830 | SRS016753 | M | 6603 | 40345 |
| SAMN00032676 | SRX023246 | SRR059854 | SRS011239 | F | 7731 | 51807 |
| SAMN00038525 | SRX023276 | SRR059884 | SRS017307 | M | 6897 | 4131 |
| SAMN00038739 | SRX023278 | SRR059886 | SRS017521 | F | 6645 | 44396 |
| SAMN00034376 | SRX023288 | SRR059896 | SRS013158 | M | 7083 | 47980 |
| SAMN00039645 | SRX023308 | SRR059916 | SRS018427 | M | 6639 | 43263 |
| SAMN00037713 | SRX023394 | SRR060025 | SRS016495 | F | 5931 | 3945 |
| SAMN00038409 | SRX023553 | SRR060358 | SRS017191 | M | 5319 | 35266 |
| SAMN00038321 | SRX023559 | SRR060364 | SRS017103 | M | 6749 | 45060 |
| SAMN00032498 | SRX023565 | SRR060370 | SRS011061 | F | 7082 | 46299 |
| SAMN00034905 | SRX023569 | SRR060374 | SRS013687 | M | 7114 | 47314 |
| SAMN00032739 | SRX023605 | SRR060410 | SRS011302 | F | 7387 | 4616 |
| SAMN00032571 | SRX023637 | SRR060442 | SRS011134 | M | 7434 | 4599 |
| SAMN00035901 | SRX023966 | SRR061135 | SRS014683 | M | 5451 | 2710 |
| SAMN00036283 | SRX023969 | SRR062320 | SRS015065 | F | 4087 | 2611 |
| SAMN00035677 | SRX023970 | SRR062321 | SRS014459 | F | 3348 | 2023 |
| SAMN00040248 | SRX023971 | SRR061140 | SRS019030 | M | 6036 | 3095 |
| SAMN00035169 | SRX023972 | SRR062323 | SRS013951 | M | 3955 | 2434 |
| SAMN00040379 | SRX023973 | SRR062324 | SRS019161 | M | 4147 | 2666 |
| SAMN00036408 | SRX023981 | SRR061147 | SRS015190 | F | 7083 | 4394 |
| SAMN00033023 | SRX023982 | SRR061144 | SRS011586 | F | 6925 | 4267 |
| SAMN00036197 | SRX023983 | SRR061149 | SRS014979 | F | 5413 | 3209 |
| SAMN00040485 | SRX023984 | SRR061150 | SRS019267 | F | 6244 | 3884 |
| SAMN00032966 | SRX023987 | SRR061153 | SRS011529 | M | 6909 | 4243 |
| SAMN00037553 | SRX023989 | SRR061161 | SRS016335 | M | 6643 | 4074 |
| SAMN00032842 | SRX023992 | SRR061164 | SRS011405 | F | 6631 | 4023 |
| SAMN00032708 | SRX023994 | SRR061168 | SRS011271 | M | 6780 | 4199 |
| SAMN00037313 | SRX023995 | SRR061172 | SRS016095 | F | 5904 | 3629 |
| SAMN00036351 | SRX024000 | SRR061204 | SRS015133 | F | 5475 | 3346 |
| SAMN00037000 | SRX024001 | SRR061208 | SRS015782 | M | 5434 | 3168 |
| SAMN00037012 | SRX024003 | SRR061185 | SRS015794 | M | 6051 | 3636 |
| SAMN00037236 | SRX024008 | SRR061197 | SRS016018 | M | 4530 | 2558 |
| SAMN00037274 | SRX024013 | SRR061198 | SRS016056 | M | 4500 | 2491 |
| SAMN00036881 | SRX024017 | SRR061205 | SRS015663 | M | 5539 | 3252 |
| SAMN00036796 | SRX024024 | SRR061222 | SRS015578 | M | 6087 | 3619 |
| SAMN00035831 | SRX024028 | SRR061226 | SRS014613 | F | 5645 | 3382 |
| SAMN00040599 | SRX024029 | SRR061256 | SRS019381 | F | 5096 | 3092 |
| SAMN00036587 | SRX024032 | SRR061254 | SRS015369 | F | 5605 | 3190 |
| SAMN00037178 | SRX024043 | SRR061255 | SRS015960 | M | 5455 | 3252 |
| SAMN00037421 | SRX024053 | SRR062538 | SRS016203 | F | 1499 | 798 |
| SAMN00036141 | SRX024084 | SRR061368 | SRS014923 | M | 5927 | 3549 |
| SAMN00036435 | SRX024088 | SRR061334 | SRS015217 | F | 5454 | 3163 |
| SAMN00040615 | SRX024148 | SRR061459 | SRS019397 | M | 5814 | 3539 |
| SAMN00037485 | SRX024165 | SRR061494 | SRS016267 | M | 5588 | 3401 |
| SAMN00037072 | SRX024166 | SRR061496 | SRS015854 | M | 5600 | 3411 |
| SAMN00039874 | SRX024173 | SRR346691 | SRS018656 | F | 6894 | 4258 |
| SAMN00035453 | SRX024198 | SRR061557 | SRS014235 | M | 5228 | 3102 |
| SAMN00039793 | SRX024203 | SRR061568 | SRS018575 | M | 4908 | 2937 |
| SAMN00032521 | SRX024515 | SRR061903 | SRS011084 | M | 7466 | 4603 |
| SAMN00038172 | SRX024531 | SRR061919 | SRS016954 | M | 7221 | 4363 |
| SAMN00037735 | SRX024545 | SRR061933 | SRS016517 | F | 7048 | 4213 |
| SAMN00039351 | SRX024585 | SRR061973 | SRS018133 | M | 6647 | 4041 |
| SAMN00039531 | SRX024623 | SRR062011 | SRS018313 | M | 7659 | 4538 |
| SAMN00032889 | SRX024944 | SRR062426 | SRS011452 | M | 2818 | 1644 |
| SAMN00035505 | SRX024954 | SRR062386 | SRS014287 | M | 4552 | 2604 |
| SAMN00040035 | SRX024963 | SRR062350 | SRS018817 | M | 6193 | 3788 |
| SAMN00035531 | SRX024982 | SRR063468 | SRS014313 | F | 5616 | 3357 |
| SAMN00036482 | SRX025177 | SRR063480 | SRS015264 | M | 3383 | 1900 |
| SAMN00034434 | SRX025444 | SRR063801 | SRS013216 | M | 758 | 1690 |
| SAMN00038208 | SRX025493 | SRR063899 | SRS016990 | M | 723 | 1624 |
| SAMN00040800 | SRX098562 | SRR346696 | SRS019582 | M | 5294 | 3070 |
| SAMN00034067 | SRX171247 | SRR528038 | SRS012849 | M | 691 | 430 |
| SAMN00037656 | SRX171266 | SRR527906 | SRS016438 | M | 889 | 585 |
| SAMN00034187 | SRX171295 | SRR528487 | SRS012969 | M | 925 | 609 |
| SAMN00034857 | SRX172903 | SRR534430 | SRS013639 | M | 680 | 459 |
| SAMN00039134 | SRX173332 | SRR541344 | SRS017916 | M | 432 | 232 |
| SAMN00034316 | SRX173450 | SRR532178 | SRS013098 | F | 404 | 269 |
| SAMN00072036 | SRX022892 | SRR059330 | SRS052027 | M | 7062 | 43981 |
| SAMN00045653 | SRX022904 | SRR059342 | SRS024435 | M | 6808 | 4184 |
| SAMN00045606 | SRX022906 | SRR059344 | SRS024388 | F | 7964 | 51281 |
| SAMN00041451 | SRX022912 | SRR059350 | SRS020233 | M | 7546 | 48301 |
| SAMN00042087 | SRX022918 | SRR059356 | SRS020869 | M | 6796 | 3974 |
| SAMN00045767 | SRX022950 | SRR059388 | SRS024549 | M | 5819 | 37480 |
| SAMN00041186 | SRX022968 | SRR059406 | SRS019968 | F | 6342 | 3848 |
| SAMN00044744 | SRX022978 | SRR059416 | SRS023526 | M | 6199 | 41558 |
| SAMN00044801 | SRX022984 | SRR059422 | SRS023583 | F | 5554 | 37044 |
| SAMN00043166 | SRX022986 | SRR059424 | SRS021948 | F | 5719 | 38386 |
| SAMN00041546 | SRX023002 | SRR059440 | SRS020328 | F | 6109 | 3645 |
| SAMN00043289 | SRX023016 | SRR059454 | SRS022071 | F | 5604 | 36110 |
| SAMN00045227 | SRX023022 | SRR059460 | SRS024009 | F | 5754 | 37716 |
| SAMN00045350 | SRX023068 | SRR059506 | SRS024132 | M | 6498 | 44658 |
| SAMN00077726 | SRX023204 | SRR059818 | SRS057717 | F | 9517 | 6088 |
| SAMN00042702 | SRX023244 | SRR059852 | SRS021484 | M | 7074 | 47689 |
| SAMN00045549 | SRX023282 | SRR059890 | SRS024331 | M | 6965 | 46098 |
| SAMN00044394 | SRX023292 | SRR059900 | SRS023176 | M | 7231 | 49104 |
| SAMN00045189 | SRX023302 | SRR059910 | SRS023971 | M | 7266 | 47902 |
| SAMN00063710 | SRX023306 | SRR059914 | SRS043701 | M | 6777 | 44575 |
| SAMN00043355 | SRX023333 | SRR059952 | SRS022137 | F | 3354 | 1541 |
| SAMN00070004 | SRX023365 | SRR059984 | SRS049995 | F | 6093 | 4037 |
| SAMN00073223 | SRX023368 | SRR059990 | SRS053214 | F | 4445 | 2848 |
| SAMN00075991 | SRX023369 | SRR059992 | SRS055982 | F | 5480 | 2583 |
| SAMN00082064 | SRX023375 | SRR060003 | SRS063040 | F | 11010 | 6980 |
| SAMN00044564 | SRX023378 | SRR060006 | SRS023346 | F | 8063 | 4655 |
| SAMN00043742 | SRX023391 | SRR060021 | SRS022524 | F | 2201 | 1179 |
| SAMN00043931 | SRX023401 | SRR060035 | SRS022713 | F | 8832 | 5624 |
| SAMN00077487 | SRX023405 | SRR060039 | SRS057478 | F | 9679 | 6119 |
| SAMN00065654 | SRX023410 | SRR060044 | SRS045645 | M | 10399 | 6516 |
| SAMN00065013 | SRX023487 | SRR060152 | SRS045004 | M | 12081 | 7315 |
| SAMN00043827 | SRX023547 | SRR060352 | SRS022609 | M | 6849 | 44221 |
| SAMN00078779 | SRX023551 | SRR060356 | SRS058770 | M | 7082 | 45770 |
| SAMN00045132 | SRX023557 | SRR060362 | SRS023914 | M | 7222 | 48246 |
| SAMN00045483 | SRX023563 | SRR060368 | SRS024265 | F | 7104 | 45924 |
| SAMN00073344 | SRX023985 | SRR061151 | SRS053335 | F | 6051 | 3671 |
| SAMN00084528 | SRX023986 | SRR061152 | SRS065504 | F | 6319 | 3880 |
| SAMN00069721 | SRX023993 | SRR061165 | SRS049712 | M | 6351 | 3908 |
| SAMN00083300 | SRX023999 | SRR061181 | SRS064276 | F | 4609 | 2646 |
| SAMN00062637 | SRX024002 | SRR061184 | SRS042628 | M | 5989 | 3668 |
| SAMN00074599 | SRX024005 | SRR061187 | SRS054590 | F | 3103 | 1745 |
| SAMN00063420 | SRX024006 | SRR061188 | SRS043411 | F | 3509 | 1938 |
| SAMN00069968 | SRX024021 | SRR061215 | SRS049959 | M | 5897 | 3682 |
| SAMN00071040 | SRX024023 | SRR061218 | SRS051031 | M | 5755 | 3478 |
| SAMN00076268 | SRX024033 | SRR061236 | SRS056259 | M | 6158 | 3860 |
| SAMN00072706 | SRX024059 | SRR061280 | SRS052697 | F | 4750 | 2676 |
| SAMN00068173 | SRX024087 | SRR061330 | SRS048164 | M | 4751 | 2773 |
| SAMN00067053 | SRX024135 | SRR061432 | SRS047044 | M | 4785 | 2763 |
| SAMN00070934 | SRX024142 | SRR061442 | SRS050925 | M | 4999 | 3014 |
| SAMN00070431 | SRX024160 | SRR061484 | SRS050422 | M | 4878 | 2898 |
| SAMN00040903 | SRX024168 | SRR061499 | SRS019685 | M | 6101 | 3623 |
| SAMN00041005 | SRX024170 | SRR061503 | SRS019787 | M | 5865 | 3465 |
| SAMN00063010 | SRX024197 | SRR061555 | SRS043001 | F | 6541 | 4114 |
| SAMN00074965 | SRX024204 | SRR061570 | SRS054956 | F | 5055 | 3021 |
| SAMN00065722 | SRX024206 | SRR061572 | SRS045713 | M | 6193 | 3787 |
| SAMN00073407 | SRX024213 | SRR061581 | SRS053398 | F | 5438 | 3271 |
| SAMN00045293 | SRX024327 | SRR061689 | SRS024075 | M | 4035 | 2499 |
| SAMN00045843 | SRX024541 | SRR061929 | SRS024625 | M | 6455 | 3832 |
| SAMN00045047 | SRX024543 | SRR061931 | SRS023829 | F | 6854 | 4206 |
| SAMN00069173 | SRX024955 | SRR062341 | SRS049164 | M | 4323 | 2517 |
| SAMN00071891 | SRX024951 | SRR062348 | SRS051882 | F | 5358 | 3208 |
| SAMN00068879 | SRX024945 | SRR062376 | SRS048870 | F | 5498 | 3349 |
| SAMN00081451 | SRX024942 | SRR062395 | SRS062427 | M | 5950 | 3633 |
| SAMN00041128 | SRX024925 | SRR062404 | SRS019910 | M | 4558 | 2615 |
| SAMN00070308 | SRX024930 | SRR062406 | SRS050299 | M | 5353 | 3200 |
| SAMN00083581 | SRX024937 | SRR062417 | SRS064557 | F | 6349 | 3840 |
| SAMN00070761 | SRX024917 | SRR062446 | SRS050752 | F | 5716 | 3427 |
| SAMN00083009 | SRX024897 | SRR062466 | SRS063985 | F | 5401 | 3267 |
| SAMN00078732 | SRX024983 | SRR063470 | SRS058723 | F | 5596 | 3326 |
| SAMN00067023 | SRX025179 | SRR063483 | SRS047014 | M | 5394 | 3221 |
| SAMN00040819 | SRX025188 | SRR063502 | SRS019601 | F | 6480 | 3914 |
| SAMN00076528 | SRX025189 | SRR063503 | SRS056519 | M | 6183 | 3731 |
| SAMN00062293 | SRX025194 | SRR063513 | SRS042284 | F | 6355 | 3844 |
| SAMN00083669 | SRX025492 | SRR063897 | SRS064645 | M | 703 | 1568 |
| SAMN00043311 | SRX025494 | SRR063901 | SRS022093 | F | 658 | 1462 |
| SAMN00069909 | SRX027122 | SRR066421 | SRS049900 | M | 14057 | 8450 |
| SAMN00042371 | SRX171345 | SRR527867 | SRS021153 | F | 620 | 383 |
| SAMN00041840 | SRX171262 | SRR527873 | SRS020622 | M | 765 | 472 |
| SAMN00070035 | SRX171284 | SRR527874 | SRS050026 | F | 690 | 448 |
| SAMN00076282 | SRX171281 | SRR527876 | SRS056273 | M | 814 | 509 |
| SAMN00083997 | SRX171338 | SRR527881 | SRS064973 | M | 709 | 439 |
| SAMN00082513 | SRX171342 | SRR527883 | SRS063489 | F | 500 | 311 |
| SAMN00045881 | SRX171255 | SRR527884 | SRS024663 | M | 754 | 493 |
| SAMN00074361 | SRX171310 | SRR527887 | SRS054352 | M | 741 | 464 |
| SAMN00084837 | SRX171304 | SRR527889 | SRS074670 | M | 721 | 445 |
| SAMN00063676 | SRX171270 | SRR527893 | SRS043667 | M | 736 | 483 |
| SAMN00069411 | SRX171303 | SRR527895 | SRS049402 | F | 639 | 403 |
| SAMN00073582 | SRX171328 | SRR527897 | SRS053573 | M | 658 | 410 |
| SAMN00073658 | SRX171294 | SRR527898 | SRS053649 | F | 728 | 481 |
| SAMN00073365 | SRX171341 | SRR527903 | SRS053356 | M | 521 | 345 |
| SAMN00075542 | SRX171246 | SRR527904 | SRS055533 | M | 809 | 501 |
| SAMN00065748 | SRX171292 | SRR527908 | SRS045739 | M | 814 | 508 |
| SAMN00085131 | SRX171320 | SRR527911 | SRS074964 | M | 856 | 536 |
| SAMN00078079 | SRX171322 | SRR527917 | SRS058070 | M | 745 | 466 |
| SAMN00042437 | SRX171317 | SRR527948 | SRS021219 | F | 997 | 625 |
| SAMN00065537 | SRX172852 | SRR531256 | SRS045528 | F | 384 | 239 |
| SAMN00082542 | SRX173404 | SRR532642 | SRS063518 | F | 757 | 508 |
| SAMN00037655 | SRX192007 | SRR642021 | SRS016437 | M | 4539 | 2835 |
| SAMN00034856 | SRX192018 | SRR646438 | SRS013638 | M | 3470 | 2159 |
| SAMN00069905 | SRX173461 | SRR532157 | SRS049896 | M | 404 | 253 |
| SAMN00088343 | SRX023373 | SRR060000 | SRS078176 | F | 4329 | 2009 |
| SAMN00087897 | SRX023472 | SRR060137 | SRS077730 | F | 5416 | 3241 |
| SAMN00085565 | SRX025503 | SRR063915 | SRS075398 | F | 6576 | 4001 |
| SAMN00086130 | SRX153910 | SRR511294 | SRS075963 | F | 804 | 559 |
| SAMN00143723 | SRX154190 | SRR511923 | SRS144506 | M | 360 | 250 |
| SAMN00087461 | SRX154177 | SRR512084 | SRS077294 | M | 8289 | 5091 |
| SAMN00143754 | SRX153886 | SRR512768 | SRS144537 | M | 13797 | 9218 |
| SAMN00087502 | SRX153894 | SRR513153 | SRS077335 | F | 11215 | 7202 |
| SAMN00099822 | SRX154187 | SRR513158 | SRS104197 | F | 18092 | 12032 |
| SAMN00087669 | SRX154173 | SRR513163 | SRS077502 | M | 10482 | 6827 |
| SAMN00142997 | SRX153970 | SRR513166 | SRS143780 | F | 20629 | 14151 |
| SAMN00095035 | SRX154180 | SRR513175 | SRS098514 | F | 19273 | 12696 |
| SAMN00142287 | SRX154071 | SRR513371 | SRS143070 | M | 9250 | 5811 |
| SAMN00095165 | SRX153900 | SRR513441 | SRS098644 | F | 16128 | 10704 |
| SAMN00143579 | SRX153975 | SRR513443 | SRS144362 | M | 17679 | 12181 |
| SAMN00142559 | SRX154203 | SRR513789 | SRS143342 | F | 13450 | 8374 |
| SAMN00099936 | SRX153918 | SRR513827 | SRS104311 | F | 15772 | 10443 |
| SAMN00087096 | SRX154097 | SRR513830 | SRS076929 | F | 10981 | 7329 |
| SAMN00142107 | SRX153914 | SRR514179 | SRS142890 | M | 10526 | 6784 |
| SAMN00143208 | SRX154219 | SRR514187 | SRS143991 | M | 10924 | 7438 |
| SAMN00087719 | SRX154102 | SRR514195 | SRS077552 | M | 20581 | 14124 |
| SAMN00143093 | SRX154238 | SRR514196 | SRS143876 | M | 21930 | 15071 |
| SAMN00141816 | SRX154127 | SRR514200 | SRS142599 | F | 12827 | 8818 |
| SAMN00095092 | SRX153934 | SRR514212 | SRS098571 | F | 16652 | 11119 |
| SAMN00031604 | SRX153932 | SRR514214 | SRS105153 | F | 18907 | 12560 |
| SAMN00142815 | SRX154000 | SRR514220 | SRS143598 | F | 17581 | 11766 |
| SAMN00087253 | SRX153938 | SRR514226 | SRS077086 | M | 9910 | 6153 |
| SAMN00094410 | SRX154092 | SRR514230 | SRS097889 | F | 18339 | 12634 |
| SAMN00087361 | SRX154095 | SRR514233 | SRS077194 | F | 16879 | 11037 |
| SAMN00141720 | SRX153999 | SRR514242 | SRS142503 | F | 17442 | 11478 |
| SAMN00142398 | SRX154103 | SRR514251 | SRS143181 | M | 11339 | 7532 |
| SAMN00095238 | SRX154224 | SRR514256 | SRS098717 | M | 12217 | 8083 |
| SAMN00099612 | SRX154109 | SRR514265 | SRS103987 | F | 22610 | 16144 |
| SAMN00141722 | SRX154018 | SRR514269 | SRS142505 | F | 9528 | 6117 |
| SAMN00100110 | SRX153920 | SRR514303 | SRS104485 | F | 18830 | 12422 |
| SAMN00142634 | SRX154017 | SRR514305 | SRS143417 | M | 11681 | 7516 |
| SAMN00141929 | SRX154234 | SRR514324 | SRS142712 | F | 10555 | 6613 |
| SAMN00100025 | SRX154242 | SRR514839 | SRS104400 | F | 17520 | 12050 |
| SAMN00095348 | SRX154229 | SRR520266 | SRS098827 | F | 12925 | 8303 |
| SAMN00097897 | SRX171319 | SRR527869 | SRS101376 | M | 616 | 385 |
| SAMN00088832 | SRX171305 | SRR527871 | SRS078665 | F | 819 | 512 |
| SAMN00097954 | SRX171286 | SRR527877 | SRS101433 | F | 450 | 282 |
| SAMN00088016 | SRX171267 | SRR527878 | SRS077849 | M | 622 | 409 |
| SAMN00088586 | SRX171248 | SRR527890 | SRS078419 | M | 855 | 534 |
| SAMN00139730 | SRX171307 | SRR527891 | SRS140513 | F | 791 | 489 |
| SAMN00139709 | SRX171279 | SRR527892 | SRS140492 | F | 901 | 569 |
| SAMN00085245 | SRX171299 | SRR527901 | SRS075078 | M | 725 | 479 |
| SAMN00088409 | SRX171272 | SRR527905 | SRS078242 | F | 738 | 463 |
| SAMN00085508 | SRX171288 | SRR527907 | SRS075341 | M | 561 | 370 |
| SAMN00139862 | SRX171265 | SRR527919 | SRS140645 | F | 1207 | 623 |
| SAMN00144714 | SRX171260 | SRR527928 | SRS145497 | F | 726 | 454 |
| SAMN00096542 | SRX171336 | SRR527942 | SRS100021 | M | 727 | 455 |
| SAMN00087920 | SRX173552 | SRR532541 | SRS077753 | F | 450 | 307 |
| SAMN00146662 | SRX153908 | SRR511293 | SRS147445 | F | 1268 | 899 |
| SAMN00145981 | SRX154079 | SRR511749 | SRS146764 | M | 212 | 147 |
| SAMN00146305 | SRX153990 | SRR511753 | SRS147088 | M | 1852 | 1171 |
| SAMN00147938 | SRX153960 | SRR512794 | SRS148721 | M | 15350 | 10918 |
| SAMN00146563 | SRX154195 | SRR513170 | SRS147346 | M | 18594 | 12655 |
| SAMN00147641 | SRX154066 | SRR513174 | SRS148424 | F | 10544 | 6545 |
| SAMN00146030 | SRX153893 | SRR513375 | SRS146813 | F | 10044 | 7268 |
| SAMN00146983 | SRX154183 | SRR513378 | SRS147766 | F | 26457 | 17942 |
| SAMN00147136 | SRX153902 | SRR513442 | SRS147919 | F | 17666 | 11432 |
| SAMN00146356 | SRX153996 | SRR514182 | SRS147139 | F | 18871 | 12853 |
| SAMN00146029 | SRX154004 | SRR514185 | SRS146812 | M | 12287 | 7710 |
| SAMN00146869 | SRX153937 | SRR514192 | SRS147652 | F | 16542 | 10499 |
| SAMN00147413 | SRX154232 | SRR514193 | SRS148196 | F | 11894 | 7931 |
| SAMN00146239 | SRX153985 | SRR514206 | SRS147022 | M | 19036 | 12904 |

**Supplementary Table S3**.List of samples in the Crohn's cohort of the HMP. (Bioproject PRJNA46321 - stool samples)

| **BioSample** | **Experiment** | **Run** | **SRA_Sample** | **sex** | **Mbases** | **Mbytes** |
| --- | --- | --- | --- | --- | --- | --- |
| SAMN00022454 | SRX021297 | SRR053011 | SRS070526 | F | 154 | 350 |
| SAMN00022453 | SRX021299 | SRR053013 | SRS070525 | F | 204 | 470 |
| SAMN00022456 | SRX021300 | SRR053014 | SRS070528 | M | 148 | 348 |
| SAMN00022456 | SRX021301 | SRR053015 | SRS070528 | M | 181 | 428 |
| SAMN00022455 | SRX021302 | SRR053016 | SRS070527 | M | 306 | 699 |
| SAMN00022447 | SRX021303 | SRR053017 | SRS070519 | M | 216 | 482 |
| SAMN00022448 | SRX021304 | SRR053018 | SRS070520 | M | 266 | 617 |
| SAMN00022461 | SRX021306 | SRR053020 | SRS070533 | F | 170 | 389 |
| SAMN00022461 | SRX021307 | SRR053021 | SRS070533 | F | 214 | 494 |
| SAMN00022460 | SRX021308 | SRR053022 | SRS070532 | F | 287 | 656 |
| SAMN00022460 | SRX021309 | SRR053023 | SRS070532 | F | 225 | 503 |
| SAMN00022451 | SRX021310 | SRR053024 | SRS070523 | F | 290 | 661 |
| SAMN00022457 | SRX021312 | SRR053025 | SRS070529 | M | 129 | 316 |
| SAMN00022458 | SRX021313 | SRR053026 | SRS070530 | M | 215 | 486 |
| SAMN00022457 | SRX021314 | SRR053027 | SRS070529 | M | 99 | 234 |
| SAMN00022462 | SRX021315 | SRR053028 | SRS070534 | F | 290 | 660 |
| SAMN00022462 | SRX021316 | SRR053029 | SRS070534 | F | 111 | 245 |
| SAMN00022462 | SRX021317 | SRR053030 | SRS070534 | F | 34 | 78 |
| SAMN00022462 | SRX021318 | SRR053031 | SRS070534 | F | 154 | 345 |
| SAMN00022450 | SRX021319 | SRR053032 | SRS070522 | M | 271 | 605 |
| SAMN00022449 | SRX021321 | SRR053034 | SRS070521 | M | 280 | 636 |
| SAMN00022464 | SRX021322 | SRR053035 | SRS070536 | M | 229 | 522 |
| SAMN00022464 | SRX021324 | SRR053036 | SRS070536 | M | 174 | 395 |
| SAMN00022452 | SRX021311 | SRR054211 | SRS070524 | F | 615 | 1408 |
| SAMN00022454 | SRX021298 | SRR053012 | SRS070526 | F | 200 | 463 |
| SAMN00022447 | SRX021305 | SRR053019 | SRS070519 | M | 157 | 366 |
| SAMN00022450 | SRX021320 | SRR053033 | SRS070522 | M | 82 | 200 |
| SAMN00022463 | SRX021323 | SRR054212 | SRS070535 | M | 384 | 859 |
| SAMN00022438 | SRX032602 | SRR090269 | SRS070510 | F | 159 | 347 |
| SAMN00022438 | SRX032602 | SRR090271 | SRS070510 | F | 274 | 602 |
| SAMN00022438 | SRX032602 | SRR090272 | SRS070510 | F | 157 | 347 |
| SAMN00022439 | SRX032603 | SRR090273 | SRS070511 | F | 304 | 664 |
| SAMN00022439 | SRX032603 | SRR090275 | SRS070511 | F | 322 | 707 |
| SAMN00829175 | SRX147090 | SRR495448 | SRS301877 | F | 345 | 786 |
| SAMN00829175 | SRX147091 | SRR495449 | SRS301877 | F | 16049 | 11848 |
| SAMN00829163 | SRX148335 | SRR497642 | SRS301865 | F | 15022 | 11305 |
| SAMN00829163 | SRX148336 | SRR497643 | SRS301865 | F | 374 | 861 |
| SAMN00829168 | SRX148337 | SRR497644 | SRS301870 | M | 13733 | 10221 |
| SAMN00829168 | SRX148338 | SRR497645 | SRS301870 | M | 278 | 619 |
| SAMN00829170 | SRX148339 | SRR497646 | SRS301872 | F | 378 | 892 |
| SAMN00829170 | SRX148340 | SRR497647 | SRS301872 | F | 13949 | 10018 |
| SAMN00829164 | SRX148341 | SRR497648 | SRS301866 | F | 371 | 866 |
| SAMN00829165 | SRX148343 | SRR497650 | SRS301867 | F | 266 | 610 |
| SAMN00829165 | SRX148344 | SRR497651 | SRS301867 | F | 9332 | 7104 |
| SAMN00829167 | SRX148345 | SRR497652 | SRS301869 | M | 360 | 825 |
| SAMN00829167 | SRX148346 | SRR497653 | SRS301869 | M | 11232 | 8514 |
| SAMN00829166 | SRX148347 | SRR497654 | SRS301868 | M | 382 | 874 |
| SAMN00829166 | SRX148348 | SRR497655 | SRS301868 | M | 32397 | 24097 |
| SAMN00829169 | SRX148349 | SRR497656 | SRS301871 | F | 16651 | 12625 |
| SAMN00829169 | SRX148350 | SRR497657 | SRS301871 | F | 326 | 761 |
| SAMN00829177 | SRX148621 | SRR497943 | SRS301879 | F | 235 | 528 |
| SAMN00829177 | SRX148622 | SRR497944 | SRS301879 | F | 20742 | 15449 |
| SAMN00829164 | SRX148342 | SRR497649 | SRS301866 | F | 16248 | 12219 |
| SAMN00829172 | SRX148623 | SRR497945 | SRS301874 | M | 14225 | 10199 |
| SAMN00829172 | SRX148624 | SRR497946 | SRS301874 | M | 398 | 910 |
| SAMN00829173 | SRX148625 | SRR497947 | SRS301875 | M | 15215 | 11380 |
| SAMN00829173 | SRX148626 | SRR497948 | SRS301875 | M | 344 | 779 |
| SAMN00829174 | SRX148627 | SRR497949 | SRS301876 | M | 363 | 836 |
| SAMN00829174 | SRX148628 | SRR497950 | SRS301876 | M | 17764 | 13542 |
| SAMN00829176 | SRX148630 | SRR497952 | SRS301878 | F | 291 | 679 |
| SAMN00829176 | SRX148629 | SRR497951 | SRS301878 | F | 8459 | 6258 |
| SAMN00829171 | SRX152081 | SRR504938 | SRS301873 | F | 18371 | 13940 |
| SAMN00829171 | SRX152082 | SRR504939 | SRS301873 | F | 268 | 613 |
| SAMN00829176 | SRX148629 | SRR513399 | SRS301878 | F | 8018 | 5964 |

**Supplementary Table S4**.List of samples in the ulcerative colitis cohort of the HMP. (Bioproject PRJNA46881 - stool samples - 16 males and one female)

| **BioSample** | **Experiment** | **Run** | **SRA_Sample** | **Mbases** | **Mbytes** |
| --- | --- | --- | --- | --- | --- |
| SAMN00023770 | SRX022536 | SRR058718 | SRS071947 | 371 | 816 |
| SAMN00023800 | SRX022537 | SRR058719 | SRS071977 | 410 | 903 |
| SAMN00023772 | SRX022737 | SRR059126 | SRS071949 | 344 | 770 |
| SAMN00023811 | SRX022738 | SRR059127 | SRS071988 | 336 | 748 |
| SAMN00023801 | SRX022739 | SRR059128 | SRS071978 | 369 | 819 |
| SAMN00023804 | SRX022740 | SRR059129 | SRS071981 | 384 | 852 |
| SAMN00023770 | SRX023437 | SRR060107 | SRS071947 | 3364 | 1940 |
| SAMN00023778 | SRX023438 | SRR060108 | SRS071955 | 4535 | 2810 |
| SAMN00023782 | SRX023439 | SRR060109 | SRS071959 | 3609 | 2171 |
| SAMN00023789 | SRX023440 | SRR060110 | SRS071966 | 195 | 95 |
| SAMN00023787 | SRX023441 | SRR060111 | SRS071964 | 4355 | 2669 |
| SAMN00023800 | SRX023442 | SRR060112 | SRS071977 | 4119 | 2512 |
| SAMN00023801 | SRX023443 | SRR060113 | SRS071978 | 4243 | 2614 |
| SAMN00023804 | SRX023444 | SRR060114 | SRS071981 | 4329 | 2676 |
| SAMN00023811 | SRX023445 | SRR060115 | SRS071988 | 3489 | 1726 |
| SAMN00023793 | SRX023446 | SRR060116 | SRS071970 | 1650 | 807 |
| SAMN00023805 | SRX023447 | SRR060117 | SRS071982 | 3691 | 1858 |

**Supplementary Table S5.**The number of cohorts with SRA-BLAST hits (a comparison with ECmurB).

|  | **Healthy Cohort** | **Crohn's disease Cohort** | **Ulcerative Colitis Cohort** |
| --- | --- | --- | --- |
| **Genes** | **Positives** | **Positives** | **Positives** |
| ECmurB | 29 | 13 | 7 |
| AMmurB | 25 | 4 | 0 |
| usp | 13 | 6 | 6 |
| tcpC | 7 | 1 | 0 |
| cnf-1 | 2 | 4 | 0 |
| gelE | 2 | 9 | 0 |
| cif | 1 | 0 | 1 |
| clbB | 1 | 1 | 0 |
| clbN | 1 | 2 | 0 |

**Supplementary Table S6**.The number of cohorts with SRA-BLAST hits (a comparison with EFmurB).

|  | **Healthy Cohort** | **Crohn's disease Cohort** | **Ulcerative Colitis Cohort** |
| --- | --- | --- | --- |
| **Genes** | **Positives** | **Positives** | **Positives** |
| EFmurB | 8 | 12 | 1 |
| AMmurB | 25 | 4 | 0 |
| usp | 13 | 6 | 6 |
| tcpC | 7 | 1 | 0 |
| cnf-1 | 2 | 4 | 0 |
| gelE | 2 | 9 | 0 |
| cif | 1 | 0 | 1 |
| clbB | 1 | 1 | 0 |
| clbN | 1 | 2 | 0 |
